# Copy number analysis by low coverage whole genome sequencing using ultra low-input DNA from formalin-fixed paraffin embedded tumor tissue

**DOI:** 10.1186/s13073-016-0375-z

**Published:** 2016-11-15

**Authors:** Tanjina Kader, David L. Goode, Stephen Q. Wong, Jacquie Connaughton, Simone M. Rowley, Lisa Devereux, David Byrne, Stephen B. Fox, Gisela Mir Arnau, Richard W. Tothill, Ian G. Campbell, Kylie L. Gorringe

**Affiliations:** 1 Cancer Genetics Laboratory, Peter MacCallum Cancer Centre, 305 Grattan St, Melbourne, VIC Australia; 2 The Sir Peter MacCallum Department of Oncology, University of Melbourne, Melbourne, VIC Australia; 3 Bioinformatics and Cancer Genomics Laboratory, Peter MacCallum Cancer Centre, 305 Grattan St, Melbourne, VIC Australia; 4 Molecular Biomarkers and Translational Genomics Laboratory, Peter MacCallum Cancer Centre, 305 Grattan St, Melbourne, VIC Australia; 5 LifePool, Peter MacCallum Cancer Centre, 305 Grattan St, Melbourne, VIC Australia; 6 Pathology, Peter MacCallum Cancer Centre, 305 Grattan St, Melbourne, VIC Australia; 7 Molecular Genomics Core Facility, Peter MacCallum Cancer Centre, 305 Grattan St, Melbourne, VIC Australia; 8 Molecular Imaging and Targeted Therapeutics Laboratory, Peter MacCallum Cancer Centre, 305 Grattan St, Melbourne, VIC Australia; 9 Department of Pathology, University of Melbourne, Parkville, VIC Australia

**Keywords:** Low coverage whole genome sequencing (LC WGS), Low-input DNA, Copy number, Formalin-fixed paraffin-embedded (FFPE), Next generation sequencing

## Abstract

Unlocking clinically translatable genomic information, including copy number alterations (CNA), from formalin-fixed paraffin-embedded (FFPE) tissue is challenging due to low yields and degraded DNA. We describe a robust, cost-effective low-coverage whole genome sequencing (LC WGS) method for CNA detection using 5 ng of FFPE-derived DNA. CN profiles using 100 ng or 5 ng input DNA were highly concordant and comparable with molecular inversion probe (MIP) array profiles. LC WGS improved CN profiles of samples that performed poorly using MIP arrays. Our technique enables identification of driver and prognostic CNAs in archival patient samples previously deemed unsuitable for genomic analysis due to DNA limitations.

**Electronic supplementary material:**

The online version of this article (doi:10.1186/s13073-016-0375-z) contains supplementary material, which is available to authorized users.

## Background

Identifying the somatic genetic alterations underlying cancer is critical to our understanding of the disease drivers and can inform diagnosis, prognosis, and response to therapy. One of the major genetic alterations in cancer is copy number alteration (CNA), with aneuploidy and structural alterations present in most malignancies, as well as being common in precursor lesions [1]. CNAs reflect the underlying biology of a tumor [2] and, given suitable detection methods, could be used in research and potentially in clinical settings to predict patients’ response to treatment and prognosis. However, the challenge of obtaining sufficient quantity and quality of DNA from formalin-fixed paraffin-embedded (FFPE) tissue has severely limited adoption of this approach.

Since the development of comparative genomic hybridization (CGH) [3], different methods have been attempted, with varying success, to identify CNAs in FFPE-derived DNA, including array-CGH and SNP arrays. One of the most reliable approaches available to date is molecular inversion probe (MIP) technology, which can obtain high-quality CNA and genotype data from FFPE samples with less than 100 ng of input DNA [4]. Additionally, because the MIP assay detects SNPs, it can also detect allelic imbalance and loss of heterozygosity (LOH) [5]. Whole genome sequencing (WGS) at 30× coverage can also be used for CNA and LOH detection, but requires at least 100 ng of high quality input DNA and has challenges associated with cost, bioinformatics processing time, and storage of large datasets. CNA from FFPE-derived DNA using low coverage WGS (LC WGS) (0.1–2× coverage) has been reported from 100 ng to 1 μg of input DNA [6–9] although point mutations and LOH were not assayed. Each method has both common and unique issues related to the required starting DNA amount, specificity, sensitivity, genome coverage, and accuracy as well as cost.

Although the high success rate of CNA by MIP assays in FFPE-derived DNA makes it arguably the best currently used method, its application remains limited since in many research and clinical settings, obtaining 100 ng of input DNA is often unachievable, particularly for small biopsy samples and pre-cancerous lesions. This limitation is particularly relevant in an era where neoadjuvant therapies may be administered before surgery, such that obtaining sufficient pre-treatment tissue for current CNA techniques from small biopsies is not feasible, once conventional diagnostic assays have been performed. A method often used to overcome the challenge of a limiting amount of DNA is whole genome amplification (WGA) to increase the amount of starting template DNA [9]. However, this method carries the risk of introducing unintended positive and/or negative CNA during the amplification process, potentially causing misinterpretation of the CN profile [10]. Therefore, there is an urgent need to develop a technique to detect CNA with high accuracy from very limited input of FFPE-derived DNA.

The primary goal of this study was to assess the performance of LC WGS to detect CNA using an ultra-low input of FFPE-derived DNA. We investigated methods of reducing DNA input and improving performance, including WGA, pre-treatment with a DNA repair procedure, and a low-input WGS library preparation method. The optimal method was then compared to MIP arrays.

## Methods

### Tumor samples and DNA extraction

Archived FFPE pathology blocks of Merkel cell carcinoma (MCC) samples (n = 2) were obtained as previously described [6]. MCC cells from these previously analyzed samples were newly micro-dissected by the Roche Automated Tissue Dissection System (Roche) from 2–3 5-μm hematoxylin and eosin (H&E) stained sections, followed by shearing with sonication with the Covaris LE220 system. DNA was extracted using a MagAttract® HMW DNA mini Kit (Qiagen).

FFPE breast tumor samples (n = 4) were obtained for this study from the LifePool cohort (www.lifepool.org). LifePool prospectively recruits Australian female participants through the population-based mammographic screening program. Participants consent to use of their diagnostic tissue blocks for research. Ten-micron sections were H&E stained and DNA was extracted from manually needle micro-dissected cells using the Qiagen DNeasy FFPE Kit (Qiagen) as previously described [11] from both FFPE breast tumor samples and two FFPE pre-cancerous breast lesions (papilloma). The quality of DNA was assessed by a multiplex PCR assay [12] modified to include additional primer sets that produce up to 700 bp fragments from non-overlapping target sites in the *GAPDH* gene.

This study was approved by the Human Research Ethics Committee at the Peter MacCallum Cancer Centre. This study was carried out in accordance with all relevant regulations and guidelines.

### Whole genome amplification

Extracted DNA from FFPE MCC samples were amplified using GenomePlex® Complete Whole Genome Amplification (WGA) kit (Sigma-Aldrich), following the manufacturer’s instruction with several minor modifications. In brief, 50 ng of DNA was prepared in a total volume of 10 μL for the fragmentation, followed by library preparation and 14 cycles of amplification as described in the protocol. The final product was purified using QIAquick® PCR purification kit (Qiagen), followed by quantification to determine the final concentration; the yield was 2–4 μg. The average fragmentation size of WGA products was 200–300 bp. A standard human genomic DNA was used as a positive control provided with the Genome Plex WGA kit (Sigma) and a no template control was used as a negative control.

### NEB next FFPE repair

The NEB Next FFPE Repair kit (NEB M6630, New England® Biolabs Inc) was used for repairing 150 ng of total DNA, according to the manufacturer’s protocol with a minor change of eluting DNA in 30 μL instead of 40 μL. A total of 10 μL of eluted DNA (total 50 ng of repaired DNA) was used for WGA using the Sigma WGA kit as described above. The remaining 100 ng of repaired DNA was used for the library prep directly using the KAPA Hyper Prep Kit.

### KAPA Hyper library preparation

Library preparation was performed as described in the KAPA Hyper Prep Kit Illumina® platforms (KR0961-v1.14, KAPA Bio systems). Slight modifications of the manufacturer’s protocol were incorporated. Briefly, 100 ng of non-WGA or unamplified DNA (both NEB Next treated repaired DNA and untreated DNA) was sheared with sonication (Covaris S2 system) for 3 × 60 s, with the following parameters: duty cycles of 10, intensity of 5, and 200 cycles/burst.

Subsequently, libraries of the both the fragmented unamplified DNA (200–400 bp) and WGA products were created by end repair and A-tailing, adaptor ligation with a stock concentration of 15 μM adaptor, followed by library amplification of six PCR cycles and eluted in 30 μL after post-amplification clean up. The library distribution was analyzed by TapeStation 2200 (Agilent Technologies) and quantified by Qubit (Life Technologies).

### NEBNext® Ultra ™ II DNA Library Prep

Library preparation was performed from MCC samples (n = 2) with 5 ng and 20 ng input, breast tumor samples (n = 4), and pre-cancerous breast lesions (papilloma) (n = 2) with 5 ng DNA input as described in the NEBNext® Ultra ™ II DNA Library Prep Kit (NEB E7645S/L, New England BioLabs ® Inc.) with several minor modifications. In brief, DNA fragmented using the Covaris S2 in 50 μL was used for NEBNext End Prep, followed by an immediate adaptor ligation step with a 1.5 μM diluted adaptor. Clean-up of adaptor-ligated DNA without size selection was carried out followed by PCR amplification with eight cycles and ten cycles for 20 ng and 5 ng input, respectively. After adding resuspended AMPure XP Beads to the PCR products, the mixture was incubated at room temperature for at least 20 min instead of 5 min. Subsequently, after adding 33 μL elution buffer (0.1 × TE) into the beads after washing with ethanol, it was incubated for 10 min instead of 2 min. A total of 2 μL of the final 30 μL library was analyzed with the TapeStation for the size distribution.

### Low coverage whole genome sequencing

The libraries prepared by both KAPA Hyper and NEBNext kits were used for LC WGS. An Illumina Nextseq platform (NextSeq 500) (paired-end 75 bp, on a mid-output flow cell) was used to run the pooled, normalized indexed libraries according to the standard protocol. The final concentration was 2 nM pooled and diluted to 1.8 pM as the standard Illumina protocol. Sequencing of those samples led to genome coverage of 1.6–1.8 × per sample.

### Molecular inversion probe SNP arrays

The Affymetrix Molecular Inversion Probe (MIP) 330 K OncoScan array was used to analyze four breast cancer samples (version 3) and two papilloma samples (version 2) and was performed according to the manufacturer’s instructions by the Ramaciotti Centre for Genomics (version 3, NSW, Australia) or Affymetrix Inc (version 2, Santa Clara, CA, USA). DNA input was 40–100 ng for this assay.

### Data analysis

Reads were aligned with bwa mem (v0.7.12-r1039) [13] to hg19 (GRCh37) after removal of sequencing primers by cutadapt (v1.7.1) [14]. ControlFREEC (version 6.7) [15] was used to estimate copy number from the low-coverage WGS data in 50 kb windows across hg19, with default parameters, no matched normal sample and baseline ploidy set to 2. Down-sampling of bam files was performed with samtools [16].

MIP data were pre-processed by the Ramaciotti Centre for Genomics or Affymterix Inc., with tumor samples batch normalized against Affymetrix controls [11].

All sample data were imported into Nexus (BioDiscovery Inc., Hawthorne, CA, USA) and segmented using SNP-FASST, a circular binary segmentation algorithm. Copy number gains were called if the log_2_ ratio of the segment was >0.15 and losses called if < –0.15. To reduce spurious calls, the genome was masked using a list of published problematic regions, including highly repetitive centromeric regions, where DNA copy number cannot be accurately measured [8].

Total CN profile overlap analysis was performed using Partek Genome Suite (Partek Inc., St. Louis, MO, USA). CNA segments for each matched pair were imported and the “finding regions in multiple samples” tool run, matching for event type (amplification/deletion). This tool reports each CNA region shared at base-pair resolution as well as each CNA region unique to a sample. Shared CN neutral regions were calculated by subtracting the length of all shared CNAs as well as sample only CNA events from the total base pairs covered.

Median Absolute Pair-wise Difference (MAPD) score was calculated as follows: if xi: is the log_2_ ratio for marker i: then MAPD = median(|*x*_i+1_ *− x*_i_ *|,i* ordered by genomic position). This metric provides a measure of the noise of the sample that is less dependent on true biological copy number variation than, for example, standard deviation.

FREEC normalized read counts in 50 kb bins were extracted from regions called as a gain or loss by FREEC in at least one of the 5 ng, 20 ng, and 100 ng DNA inputs or the WGA libraries for MCT-4 and MCT-6 LC WGS data. Gains or losses in regions lacking MIP array probes and regions in the blacklist of Scheinin et al. [8] were filtered out. The Pearson correlation of bin counts in these CNA regions was calculated and used to cluster by Euclidean distance using the hclust() function of R 3.2.1. Correlation between samples was visualized using the pheatmap package.

## Results

### Comparing copy number alteration calls using low-input DNA

**Fig. 1:**
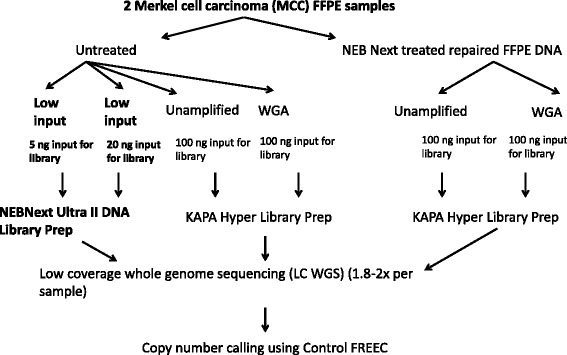
Experimental design testing a low-input method (NEBNext Ultra II) on copy number detection of two FFPE MCC samples by LC WGS as well as the effect of WGA with or without NEB DNA repair treatment

**Fig. 2:**
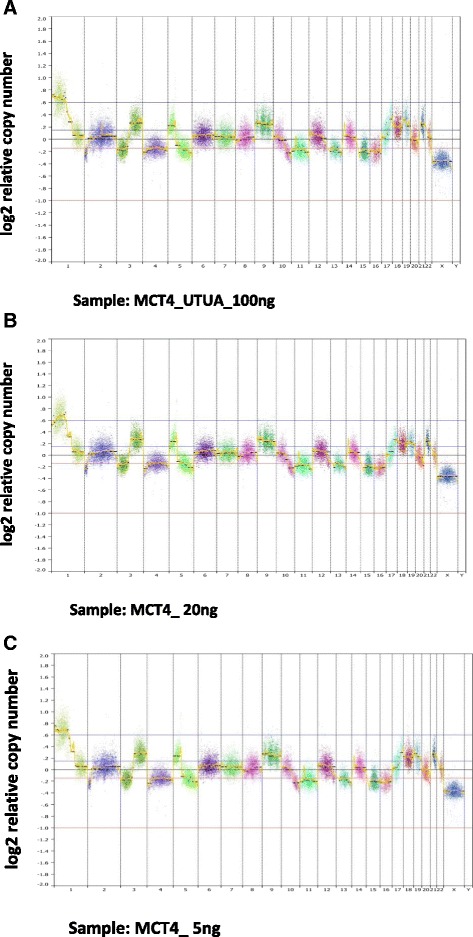
Copy number profiles of MCC sample MCT4 with DNA input of (**a**) 100 ng, (**b**) 20 ng, and (**c**) 5 ng. Each point represents the normalized read count ratio of a 50 kb sized bin. Separate chromosomes from 1 to 22 as well as X and Y are shown and a log_2_ (copy number/2) equal to zero corresponds to a copy number of 2. Segments were removed from highly repetitive or problematic regions [8]

**Fig. 3:**
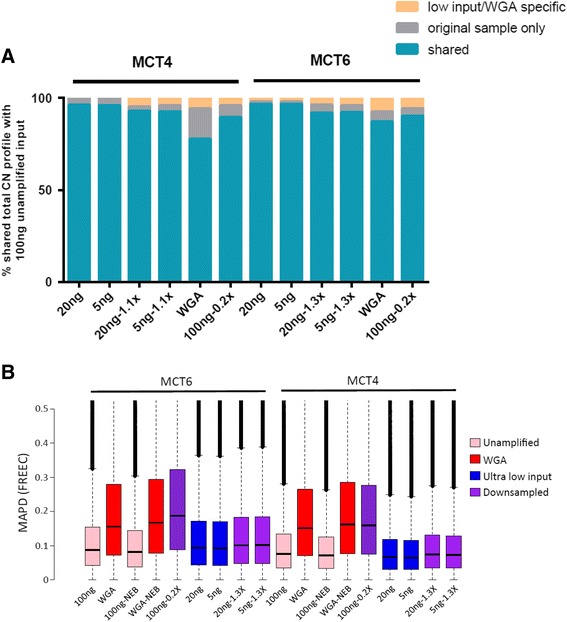
**a** Concordance in CN profiles between samples, expressed as the percentage of sites in the genome called diploid, gain, or loss concordant with 100 ng unamplified input of two MCC samples, MCT4, and MCT6. **b** Distribution of MAPD values from FREEC normalized bin counts across all samples of low input 20 ng and 5 ng samples (*blue*) and low input 20 ng and 5 ng down-sampled to 1.3×, the mean depth of the 100 ng input samples (*purple*). Whole genome amplified (WGA, *red*), Unamplified (UA, *pink*) along with NEB treated (NEB) or untreated (UT), unamplified down-sampled to 0.2×, the mean depth of the WGA samples (*purple*) are also shown (all 100 ng input to library preparation)

We investigated a recently developed library preparation method (NEBNext Ultra II) to reduce the required input of DNA (Fig. 1). DNA was obtained from two archival FFPE Merkel cell carcinoma (MCC) samples [6]. LC WGS was performed on 100 ng, 20 ng, and 5 ng input DNA. Compared with the standard 100 ng input, comparable CN profiles were observed using 5 ng or 20 ng input DNA with 95 % of CN calls (gain, loss, or no change) being concordant on average (Figs. 2 and 3). In addition, the quality control metric MAPD was comparable between the different DNA inputs (Fig. 3).

Since the low-input samples (both 20 ng and 5 ng) were sequenced at a higher mean coverage (Table 1) than the 100 ng input samples, the 20 ng and 5 ng samples were computationally down-sampled to simulate the mean coverage of the 100 ng input (1.3×). The CN profiles of the down-sampled low input samples still showed 91–93 % concordance with CN profiles from the matched 100 ng input samples (Fig. 3) with only minor increases in MAPD (Fig. 3).

**Table 1:** Sequencing performance for all samples

	MCT6-NEB-UA	MCT6-NEB-WGA	MCT6-UT-UA	MCT6-UT-WGA	MCT6-20 ng	MCT6-5 ng	MCT4-NEB-UA	MCT4-NEB-WGA	MCT4-UT-UA	MCT4-UT-WGA	MCT4-20 ng	MCT4-5 ng	LP S1	LP S2	LP S3	LP S4	P1	P2
Mean coverage	1.34	0.14	1	0.18	1.96	2.23	1.53	0.19	1.24	0.22	2.25	2.13	2.66	1.68	1.8	2.1	1.98	0.75
Total reads (millions)	62.4	16.4	48.5	17.9	90.2	104.9	72.1	19.2	59.3	19.9	101.7	101.9	119.9	81.3	88.3	96.1	88.4	40.3
Mapped reads (millions)	61.7	14.0	47.0	15.4	88.5	102.5	71.2	15.9	57.7	17.1	101.1	100.9	119.0	80.8	87.7	95.5	87.7	39.5
Reads mapped (%)	98.96	85.57	96.97	85.85	98.04	97.66	98.73	83.18	97.35	86.17	99.4	99.1	99.28	99.39	99.29	99.32	99.23	98.09
Reads duplicates (%)	4.89	35.67	5.73	26.73	4.13	5.74	5.14	28.8	5.73	20.65	4.54	8.28	5.14	7.26	10.56	5.38	4.18	12.66
Total reads minus duplicates (millions)	59.4	11.4	45.8	13.8	86.5	99.0	68.4	14.6	55.9	16.3	97.1	93.5	113.8	75.5	79.1	90.9	84.7	35.3
Target bases (%) > = onefold coverage	58.26	9.17	48.53	11.41	67.97	71.54	61.91	11.45	56.48	14.2	74.44	72.15	80.14	62.94	65.31	73.84	72.33	33.37
Target bases (%) > = tenfold coverage	0.14	0.01	0.07	0.01	0.48	0.78	0.19	0.01	0.11	0.02	0.6	0.52	0.83	0.26	0.3	0.31	0.25	0.06
Median fragment length	124	80	120	85	111	110	113	88	124	98	120	111	124	96	101	119	122	79
Tissue age (years)	5	5	5	5	5	5	5	5	5	5	5	5	2	7	2	9	7	11

*NEB* treated with NEB repair kit, *UT* untreated, *UA* unamplified, *MCT* MCC samples, *LP* breast tumor samples, *P* pre-cancerous breast lesions

### Comparison of CN profiles from low-input LC WGS and MIP arrays

**Fig. 4:**
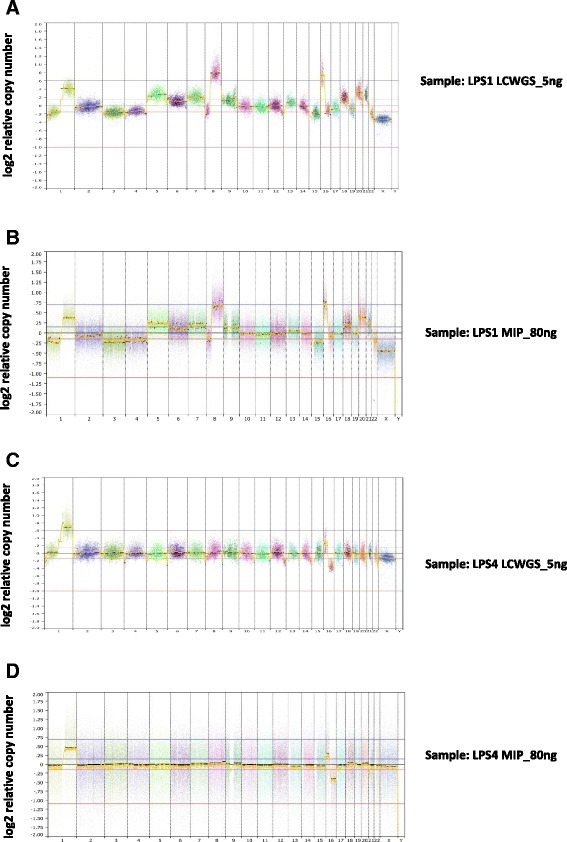
CN profiles for two breast tumor samples (LP S1 (**a**, **b**) and LP S4 (**c**, **d**)). **a**, **c** Low coverage WGS from 5 ng input of DNA. **b**, **d** MIP arrays in the range of 80–100 ng input of DNA. Each data point in (**a**) and (**c**) represents normalized read count ratios from a 50 kb window. Segments were removed from highly repetitive or problematic regions [8]

As the Affymetrix OncoScan MIP arrays are considered by many to be a high-quality method for CN analysis of FFPE samples [17], we compared the performance of low-input LC WGS against these SNP arrays using matched DNA from four FFPE breast cancer samples (LPS1-LPS4). The CN profiles derived from LC WGS were comparable to and, in some cases, improved upon MIP arrays (Fig. 4). Overall, LC WGS with 5 ng of input DNA resulted in CN profiles with >80 % (80–93 %) concordance with those produced using 80–100 ng input DNA on MIP arrays (Table 2). LC WGS typically covers 60–80 % of the sites in hg19 (Table 1), providing broader sampling of the genome, apart from the genomic regions known to be problematic for CN estimation [8], than MIP arrays, which interrogate ~330,000 selected sites that may not be distributed evenly across the genome.

**Table 2:** Concordance in CN profiles between samples, expressed as percentage of sites in the genome called diploid, gain, or loss in both the LC WGS and MIP arrays for each breast tumor sample, and the percentage of sites called as gain or loss in the LC WGS or MIP results only, respectively. Concordance in only CNV regions in both LC WGS and MIP arrays, expressed as percentage, for each breast tumor sample

Sample	Shared sites (%)	WGS only sites (%)	CNA WGS only (Mbp)	MIP only sites (%)	CNA MIP only (Mbp)	Shared CNV (%)
LPS1	80.0	10.1	263	9.9	260	68
LPS2	83.4	7.7	202	8.8	231	74
LPS3	83.0	9.6	198	7.4	136	72
LPS4	93.1	6.6	173	0.34	8.9	49

It is noteworthy that from the overlap analysis (Fig. 3), on average 15 % of the total CN profiles from LC WGS and MIP differed; these differences fell into two categories. First, in some cases, LC WGS provided higher sensitivity to detect small CN changes by providing more even coverage across the genome than SNP arrays, whereas, in other regions where SNP density was high, the MIP arrays were able to detect CNA with length <50 kb, below the detection limit imposed by the chosen window size for LC WGS analysis. Second, many of the large-scale differences were caused by segmentation and thresholding differences, rather than true CN changes (Additional file 1: Figure S1 and S2). For example, in LP S1, MIP arrays called chromosome 4 as a loss whereas no CNA was called from the LC WGS data (Additional file 1: Figure S2). However, that particular loss could be explained by some segments sitting just below the threshold in the MIP data whereas in LC WGS bins they did not, due to normalization subtly shifting read counts upward across the genome. For three samples, we had orthogonal CN data from a targeted sequencing assay. Good concordance was observed for CN variable regions between LC WGS and this assay (LPS1 84 %, LPS2 94 %, and LPS4 61 % of CNA bp concordant). The concordance between MIP arrays and the targeted assay was similar for CNA regions (87 %, 95 %, and 61 %, respectively).

**Fig. 5:**
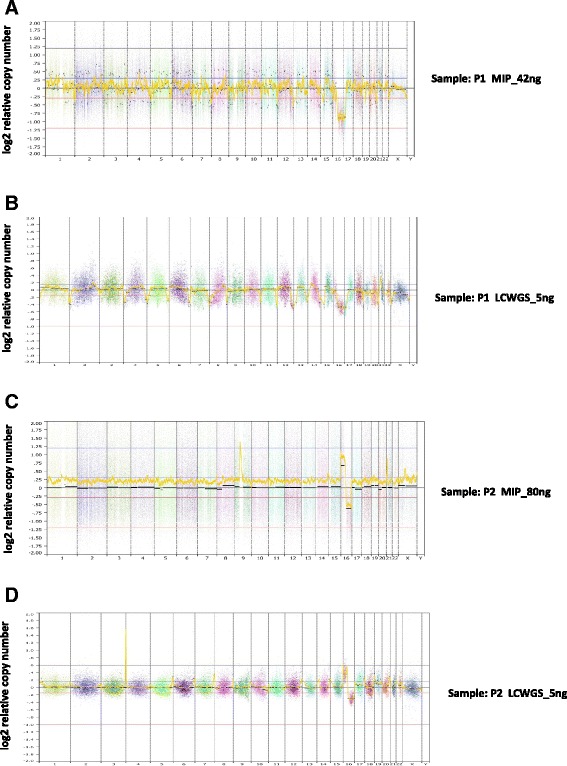
CN profiles for two breast pre-cancerous samples (P1 (**a**, **b**) and P2 (**c**, **d**)). **a**, **c** MIP arrays in the range of 40–80 ng input of DNA. **b**, **d** LC WGS from 5 ng input of DNA. Each data point in (**b**) and (**d**) represents normalized read count ratios from a 50 kb window. Segments were removed from highly repetitive or problematic regions [8]

We also compared the performance of low-input LC WGS against MIP arrays using matched DNA from two FFPE pre-cancerous breast lesions in order to investigate whether LC WGS could offer an improvement upon very poorly performed MIP assays. Both low-quality DNA LC WGS samples demonstrated improved resolution of CNAs as compared with MIP arrays (Fig. 5). P1 showed markedly improved segmentation continuity, with 1306 segments resolving into 68 segments. Sample P2 showed a particularly big reduction in bin-to-bin variability (Additional file 1: Figure S3) and the proportion of data points greater than twice the mean CNA value reduced from 17 % to just 1 %.

The FFPE repair treatment made little discernible difference to the appearance of the CN profiles (data not shown) or MAPD scores (Fig. 3), although the sequencing metrics were marginally improved compared with untreated samples (Table 1).

### Comparison of CN profiles between unamplified and WGA samples with or without NEB FFPE repair treatment

**Fig. 6:**
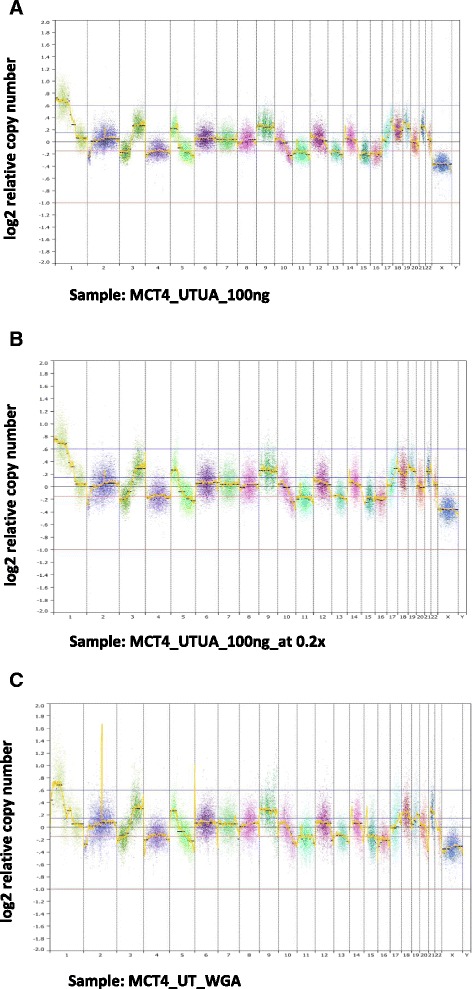
CN profiles of unamplified (UA) and WGA samples. **a** CN profile of MCC sample MCT4, which is unamplified and untreated, with 100 ng input of DNA. **b** CN profile of the same sample, which is unamplified and untreated, down-sampled to the similar coverage as the matched WGA sample. **c** CN profile of the same sample, which is WGA and untreated. Each point represents the normalized read count ratio of a 50 kb sized bin. Separate chromosomes from 1 to 22 as well as X and Y are shown and a log_2_ (copy number/2) equal to zero corresponds to a copy number of 2. Segments were removed from highly repetitive or problematic regions [8]

We additionally evaluated WGA as an alternative method of reducing the amount of native input DNA into LC WGS without compromising CNA detection. In parallel, we assessed whether a DNA repair procedure (NEB Next) could improve LC WGS CNA detection performance. The experimental strategy is summarized in Fig. 1. Fifty nanograms of DNA derived from two archival FFPE MCC samples [6] were subjected to WGA and this yielded 2–4 μg of product, indicating that WGA was successful. LC WGS was performed on the same amount of input DNA (100 ng) from unamplified and WGA samples. Compared with the unamplified samples, the WGA samples had fewer reads mapped, approximately six times as many duplicate reads, and <15 % of the genome covered by at least one read (Table 1). The poorer sequencing metrics were reflected in the CN profiles with the unamplified samples showing less variability in read counts per genomic segment and more clearly discernible CNAs (Fig. 6). Further investigation revealed the poor sequencing results from the WGA samples are mostly likely related to base calling and read mapping being compromised by the presence of adaptors from WGA primers in the reads (Additional file 1: Figure S4).

Overall, 77–87 % of the total CN profiles from matched unamplified or WGA samples were concordant (Fig. 3). Unsupervised clustering of MCT-4 and MCT-6 CNA showed high intra-sample concordance with different input amounts and methods, although the WGA data had longer branch lengths (Additional file 1: Figure S5). Variance in read distribution as calculated by MAPD was much higher in WGA samples (Fig. 3), consistent with the higher level of noise observed in CN profiles of WGA samples as compared with unamplified. Down-sampling reads from the untreated unamplified samples to coverage equivalent to the WGA samples (0.2×) revealed the reduction in consistent CNA calls from the latter could not be attributed to differences in read depth alone (Fig. 3).

## Discussion

Identifying CNAs by CGH or MIP arrays has been used successfully for DNA derived from FFPE-fixed tissue, although most often with a DNA input of >100 ng [4, 18, 19]. More recently, LC WGS has also been successfully used to assess CNAs but again mostly using at least 100 ng of input DNA with coverage of 0.1–3× [6, 8, 9]. A very recent study used a complex cell-sorting and single tube Ion Torrent amplicon-based library preparation method to obtain LC WGS CN profiles from 126–300 cells without extracting DNA [8, 9, 20]. To open CN analysis to samples with very limited DNA extracted by standard methods and compatible with the common Illumina sequencing platforms, we evaluated methods to reduce the required DNA input for LC WGS.

A recently released library preparation method (NEB Ultra II) adapted for very low input DNA was successfully tested. The novel combination of this commercially available kit and our modifications for low input DNA enabled us to obtain high-quality CN data, which was not previously possible. When we compared the CN profile and MAPD results of 5 ng, 20 ng, and 100 ng input, we observed almost negligible difference between these three inputs with 95 % overlap of CN profiles. Even down-sampling of low input samples showed almost 92 % overlap of CN calls with the matched UA-UT 100 ng input, which had lower mean coverage, without significantly changing MAPD. This result confirmed that 5 ng of input DNA produces total CN profiles that are highly concordant with those obtained from 100 ng of input DNA at the same depth of sequencing coverage. Interestingly, samples we tested with even less than 5 ng input showed on average 90 % overlap of CN calls (data not shown), however, at 2.5 ng and 1 ng inputs there were assay failures for some samples, suggesting that a novel methodology needs to be developed in future to robustly utilize DNA input lower than 5 ng. We have subsequently tested an additional 12 cases (21 DNA samples) and obtained good quality CN data from all cases using 5 ng DNA (unpublished data). Our tested cases are in the age range of 1–12 years and we found a weak trend between the age of the block and the QC score calculated by Nexus (*p* = 0.06, Kruskal–Wallis test for samples grouped in 5-year intervals, Additional file 1: Figure S6). The difference in QC score was subtle and did not affect detection of CNA.

We observed a high degree of similarity in CNAs detected by low input LC WGS and MIP arrays, despite the more than tenfold higher input DNA used for the array-based method, with a much-improved CN profile for samples that had performed poorly by MIP. Additionally, the 15 % of total CN profile dissimilarity seen on average between LC WGS and MIP arrays could be explained by more uniform coverage of LC WGS or segmentation and thresholding differences, rather than true CN changes. A limitation of the study is the lack of fresh-frozen tissue to extract high-quality DNA for comparison; however, high concordance of MIP array data between FFPE and fresh-frozen derived DNA has been demonstrated previously [4].

A limitation of the LC WGS approach is the inability to determine allelic imbalance at high resolution due to the low mean base coverage. However, in a high-quality sample, increasing the average read depth to approximately tenfold would lead to >30 % of the genome having sufficient coverage to call a genotype [21]. Even in a low-quality FFPE-derived sample with reduced call rates, this level would still be more than sufficient for detection of allelic imbalance and provide resolution similar to MIP arrays. An FFPE WGS study of two breast cancer samples identified ~2 million high-confidence SNP calls from ~20-fold coverage [22].

One common method for increasing available input DNA is WGA, which has been coupled with various array-based systems for CNA studies [9, 23]. However, in our study, WGA resulted in poor sequencing performance (Table 1) and poor overlap (77–87 %) of CNA data derived from unamplified DNA. While high concordance rates and reproducibility has been reported using WGA along with SNP genotyping [24], some published studies have suggested that false-positive CNA could be introduced randomly during the amplification process and also that use of WGA could obscure true CN changes [10, 23]. The high MAPD values from WGA input and the noise seen in the CN profiles concurs with previous array-based studies [10].

While amplification bias during WGA may account for some of our observations, the poor performance of the WGA samples in our case also stems from the incorporation of universal adaptors prior to Illumina library preparation, limiting nucleotide diversity during the first bases of sequencing and resulting in a dramatic decrease in the number and quality of sequenced fragments (Additional file 1: Figure S4). Similar results were reported previously [9]. Some of the technical challenges presented by WGA may be overcome by optimization; however, given that low input library preparation methods give reliable and accurate results, the imperative to use WGA is removed.

Interestingly, the FFPE DNA repairing method used did not show any improvement in CN profile for either amplified or unamplified samples, although the sequencing metrics were slightly better. One possible reason for observing no significant differences in CN analysis using the repair method is that while this method repairs single-strand nicks, DNA fragmentation and DNA-protein cross-links remain, likely leading to sub-optimal library preparation. This result suggested that such repair methods might not be necessary for CN analysis; however, the possibility remains that they could improve identification of other genomic alterations such as somatic point mutations.

## Conclusions

The major goal of this study was to investigate methods for achieving accurate CN detection with as little input DNA as possible. CNA are often associated with prognosis for a variety of tumor types including pre-cancerous lesions [1, 2, 25]. In situations where more than 10 ng DNA is unavailable, either in research or clinical settings, LC WGS using the low input method described here would be a highly suitable method in terms of accuracy, sensitivity, specificity, speed, and cost to detect CN changes in FFPE samples. This technique opens up the possibility of obtaining high-quality genome-wide copy number from vast archives of FFPE tissue without depleting the tissue resource, thereby enabling highly powered retrospective studies of associations of CN events with clinical features. Small, previously intractable lesions can now be investigated fully and, in addition, this technique could be developed into a clinically feasible assay that, for the same price as FISH, can interrogate the entire genome.

## Additional file

Additional file 1:
**Figure S1.** Profile of chromosome 7 for LPS1; **Figure S2.** Profile of chromosome 4 for LPS1; **Figure S3.**Comparison of measurement variability (MAPD); **Figure S4.** Alignment of reads from a WGA sample; **Figure S5.** Clustering of MCT-4 and MCT-6 5 ng, 20 ng, 100 ng (UA) and WGA; **Figure S6.** Correlation of FFPE block age with QC score. (PDF 823 kb)
