# Supplementary material for: Copy number analysis by low coverage whole genome sequencing using ultra low-input DNA from formalin-fixed paraffin embedded tumor tissue
Source: Genome Med. 2016 Nov 15;8:121. doi: 10.1186/s13073-016-0375-z (PMC5111221; doi:10.1186/s13073-016-0375-z)
Supplement: Additional file 1: — Figure S1. Profile of chromosome 7 for LPS1; Figure S2. Profile of chromosome 4 for LPS1; Figure S3.Comparison of measurement variability (MAPD); Figure S4. Alignment of reads from a WGA sample; Figure S5. Clustering of MCT-4 and MCT-6 5 ng, 20 ng, 100 ng (UA) and WGA; Figure S6. Correlation of FFPE block age with QC score. (PDF 823 kb) [file 13073_2016_375_MOESM1_ESM.pdf]

Supplementary Figure 1

A

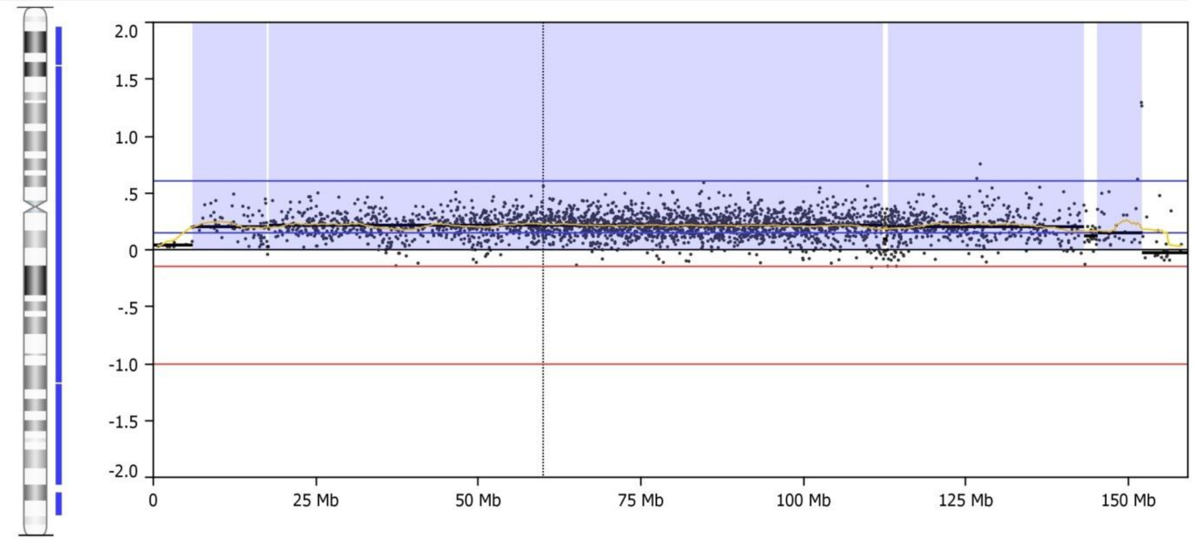

Sample: LP S1- LC WGS: Chromosome 7

B

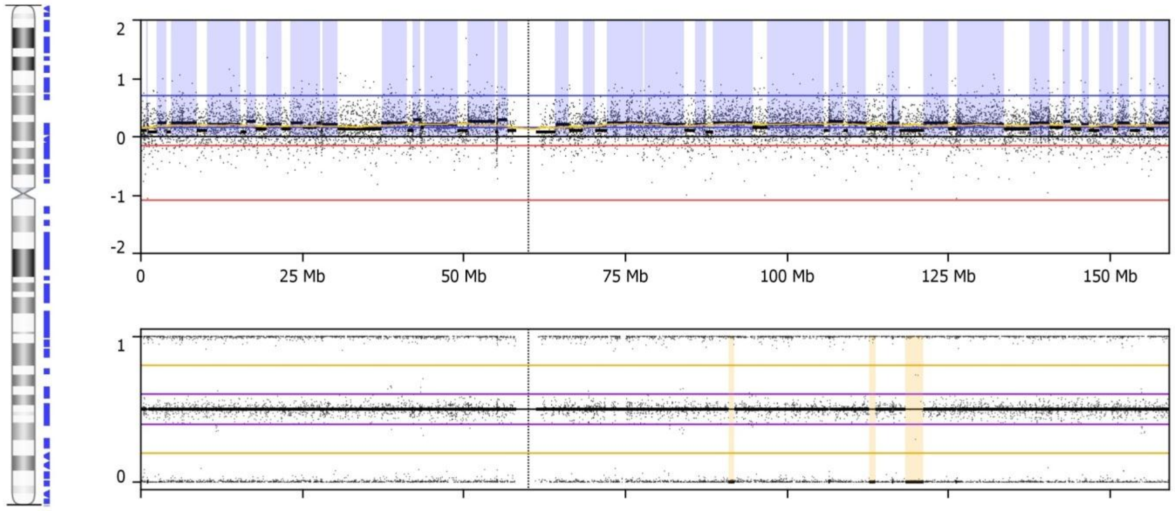

Sample: LP S1-MIP: Chromosome 7

Supplementary Figure 1. Profile of chromosome 7 for breast tumour sample (LP S1 (A, B)). (A) Low coverage WGS from 5 ng input of DNA. Each data point represents normalised read count ratios from a 50 kb window. (B) MIP arrays from 80-100 ng input of DNA. Each data point represents a single probe.

Supplementary Figure 2

A

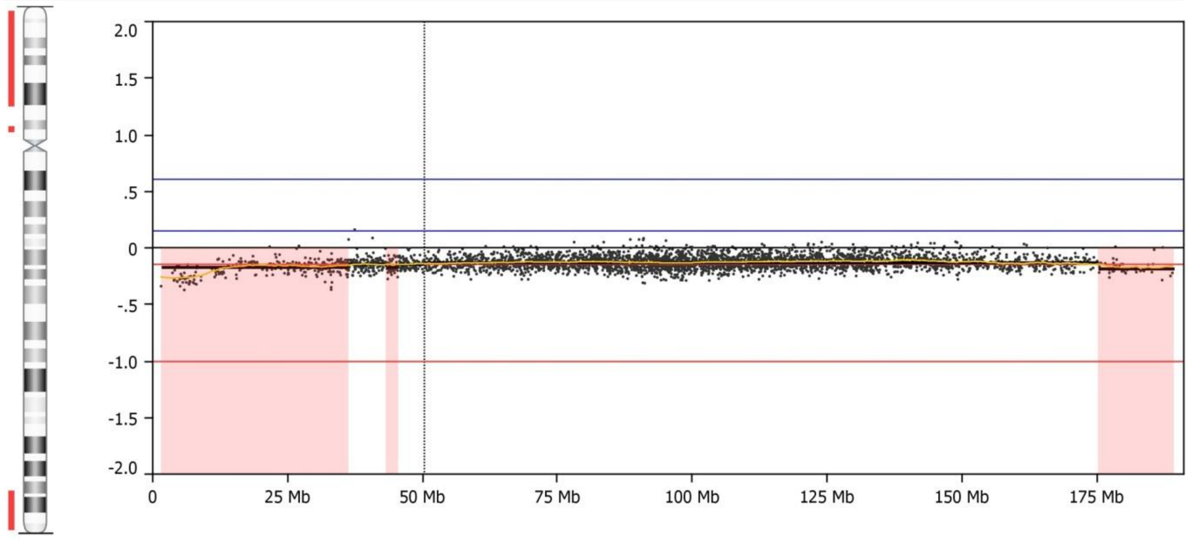

Sample: LP S1- LC WGS: Chromosome 4

B

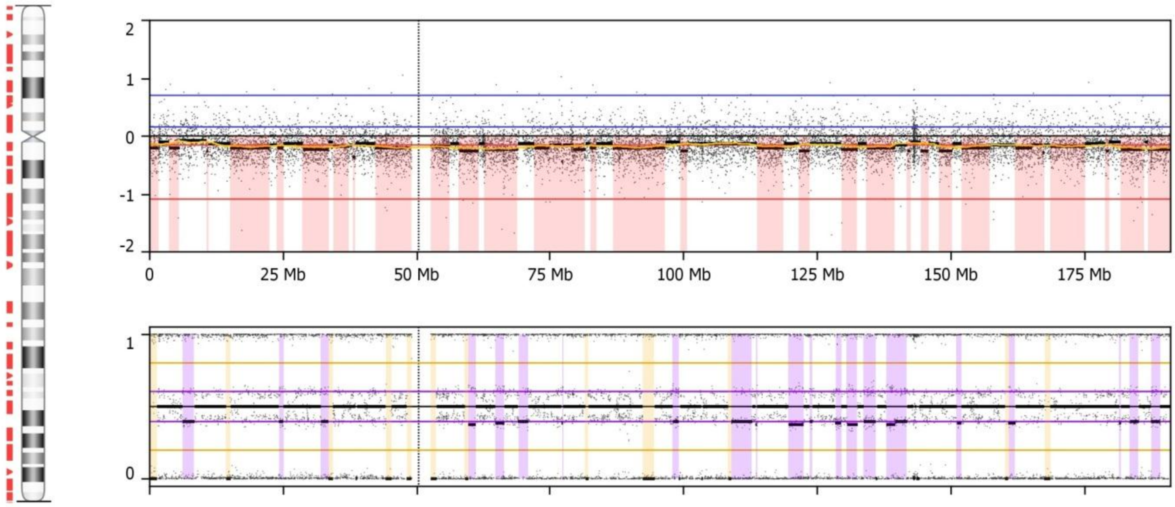

Sample: LP S1-MIP: Chromosome 4

Supplementary Figure 2. Profile of chromosome 4 for breast tumour sample (LP S1 (A, B)). (A) Low coverage WGS from 5 ng input of DNA. (B) MIP arrays from 80-100 ng input of DNA.

Supplementary Figure 3

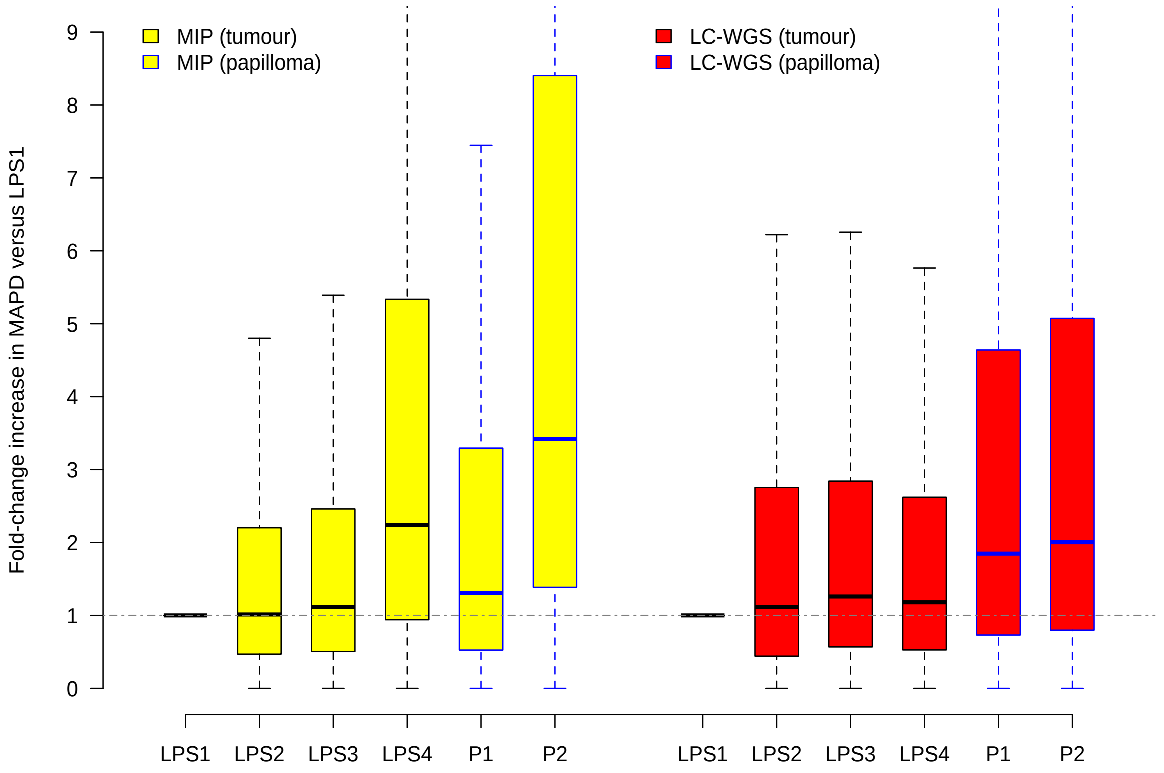

Supplementary Figure 3. Comparison of measurement variability as measured by MAPD from MIP arrays (left; yellow) and LC WGS (right; red) from high-quality DNA from breast tumour samples (black outline) and low-quality DNA from papillomas (blue outline) using a 100 ng breast tumour sample with the lowest MAPD (LPS1) as a baseline. LC-WGS demonstrates a 50% decrease in measurement noise compared to MIPs for the lowest quality sample (P2). Sample P2 has a 3-fold increase in median MAPD compared to LPS1 for the MIP array data, but with LC-WGS data the increase in MAPD is only 2-fold.

Supplementary Figure 4

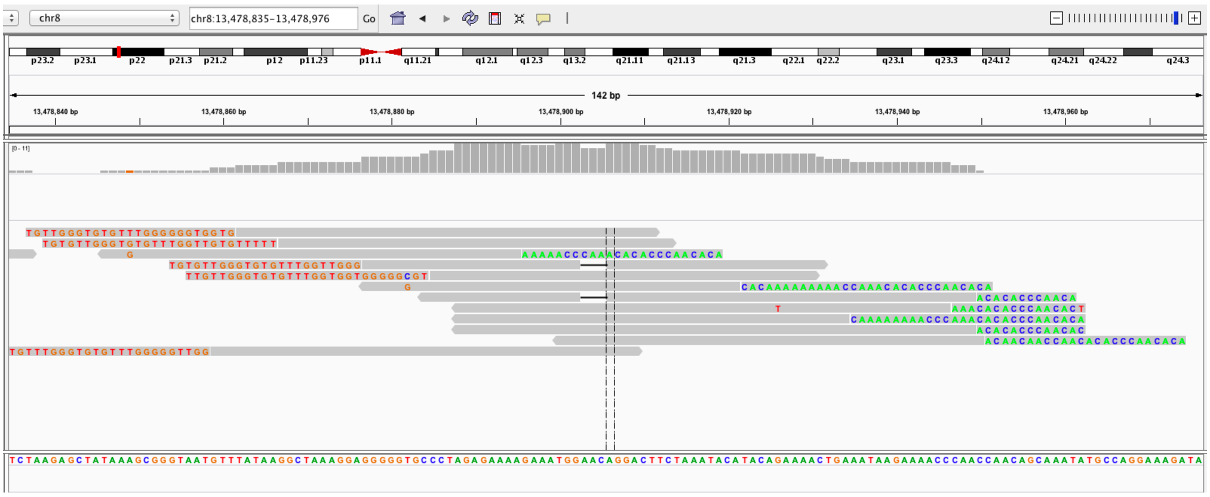

Supplementary Figure 4. Alignment of reads from a WGA sample to hg19. Note the low coverage and soft-masking of WGA adaptor ligated sequence.

Supplementary Figure 5

A

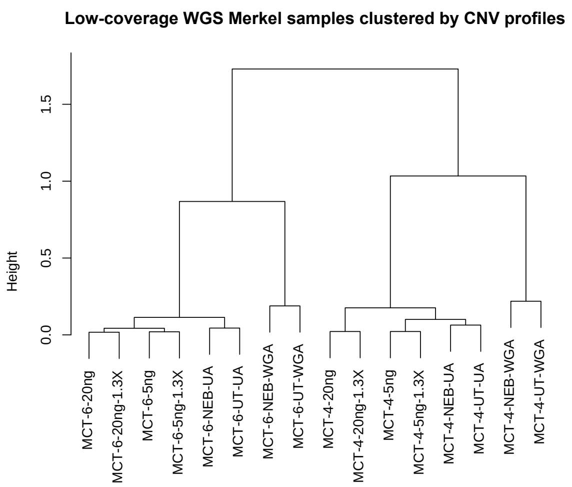

B

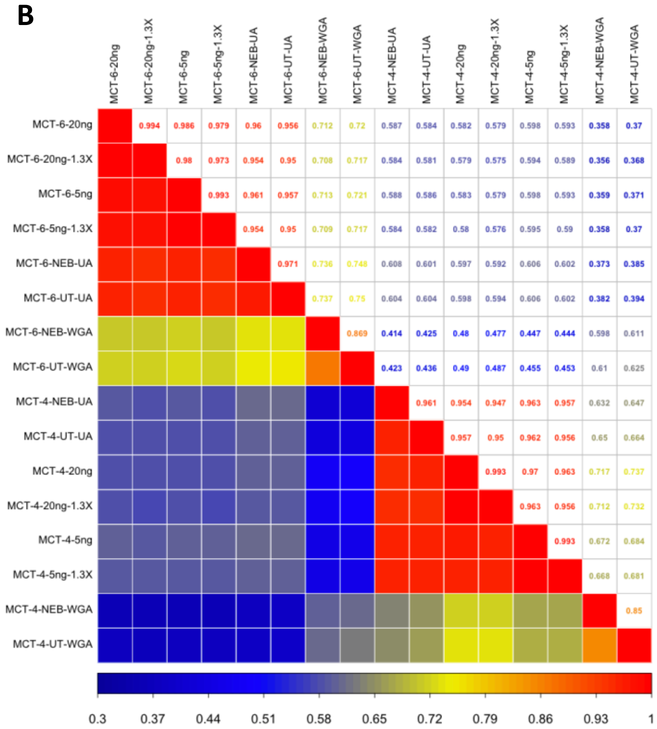

Supplementary Figure 5. Results of clustering all MCT-4 and MCT-6 5 ng, 20 ng, 100 ng (UA) and WGA samples by FREEC normalized read counts in 50kb bins in regions called as copy-number aberrant in at least one of the MCT-4 and MCT-6 samples. (A) Euclidean distance between samples. (B) Pearson correlation coefficients between pairs of samples, with red indicating high correlation (>0.9), yellow moderate correlation (0.65 – 0.9) and blue lower correlation (<0.65).

Supplementary Figure 6

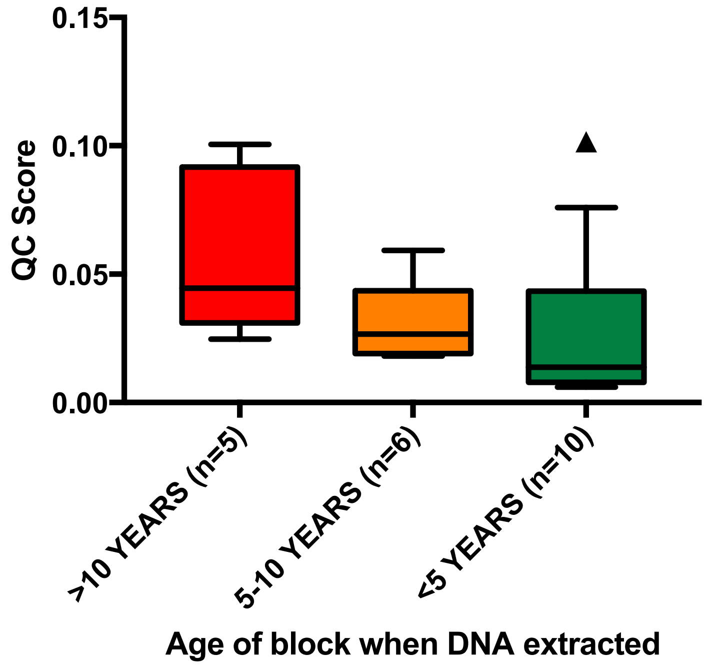

Supplementary Figure 6. Correlation of FFPE block age with Nexus QC score for LC WGS derived copy number (p=0.06, Kruskal-Wallis test for samples grouped in 5-year intervals)
